# A New Approach to Assess the Gastrocnemius Muscle Volume in Rodents Using Ultrasound; Comparison with the Gastrocnemius Muscle Index

**DOI:** 10.1371/journal.pone.0054041

**Published:** 2013-01-10

**Authors:** Tim H. J. Nijhuis, Siebe A. S. de Boer, Abhijeet L. Wahegaonkar, Allen T. Bishop, Alexander Y. Shin, Steven E. R. Hovius, Ruud W. Selles

**Affiliations:** 1 Department of Plastic, Reconstructive and Hand Surgery, Erasmus MC, University Medical Center, Rotterdam, The Netherlands; 2 Department of Orthopaedic Surgery, Division of Hand Surgery, Mayo Clinic, Rochester, Minnesota, United States of America; 3 Microvascular Research Laboratory, Mayo Clinic, Rochester, Minnesota, United States of America; Creighton University, United States of America

## Abstract

**Introduction:**

The purpose of this study was to determine the reliability and validity of a new non-invasive ultrasound technique to measure gastrocnemius muscle atrophy after nerve denervation in an animal model.

**Methods:**

In sixteen rodents an eight mm sciatic nerve gap was created. In the following 8 weeks, each week, two rodents were euthanized and the gastrocnemius muscle was examined using two different ultrasound systems and two investigators. The standardized ultrasound measurement protocol consisted of identifying pre-defined anatomical landmarks: 1) the fibula, 2) the fibular nerve, and 3) the junction between the most distal point of the semitendinosus muscle and gastrocnemius muscle. Consequently, we measured the muscle thickness as the length of the line between the fibula and the junction between the two muscles, perpendicular to the fibular nerve. After the ultrasound recording, the muscle mass was determined.

**Results:**

A steep decline of muscle weight of 24% was observed after one week. In the following weeks, the weight further decreased and then remained stable from 6 weeks onwards, resulting in a maximal muscle weight decrease of 82%. The correlation coefficient was >0.96 between muscle diameter and weight using both ultrasound systems. The inter-rater reliability was excellent for both devices on the operated side (ICC of 0.99 for both ultrasound systems) and good for the non-operated site (ICC’s: 0.84 & 0.89). The difference between the muscle mass ratio and the muscle thickness ratio was not more than 5% with two outliers of approximately 13%.

**Discussion:**

We have developed an innovative, highly reliable technique for quantifying muscle atrophy after nerve injury. This technique allows serial measurements in the same animal over time. This is a significant advantage compared to the conventional technique for quantifying muscle atrophy, which requires sacrificing the animal.

## Introduction

The preferred treatment of a peripheral nerve injury is a direct tension-free end-to-end repair. [1] However, in large nerve defects, an interposition graft is required, as tension on the nerve repair is detrimental to neural regeneration. [2], [3] A nerve autograft is considered the “Gold Standard” for these large defects, although full functional recovery is rarely achieved. [4] Therefore, innovative and more effective nerve reconstruction techniques are still sought [5], [6].

As a first step in the development of reconstruction techniques, in vivo animal experiments are widely used. [7] In these experiments, a number of different techniques are used to evaluate both functional and histological regeneration. One measure of motor outcome is the gastrocnemius muscle index (GMI), which is a standardized measure of gastrocnemius muscle weight, normalized to the contralateral gastrocnemius muscle weight. The rationale for quantifying muscle weight is that the weight decreases after denervation due to atrophy followed by an increase after re-innervation. As such, the GMI is an indirect measure for muscle force and thus nerve regeneration [8].

The GMI is calculated by dividing the wet gastrocnemius muscle weight of the operated leg with the contralateral side. Calculation of the GMI is a sacrificial procedure and does not allow serial assessment of nerve re-innervation of the gastrocnemius muscle. A significant disadvantage of the GMI calculation is therefore that a separate group of animals must be sacrificed for each specific time point to determine the pattern of atrophy and the regain of muscle weight over time. [9] As a result, a relatively lager number animals is required when the GMI is used to assess motor recovery. Repeated measurement of muscle atrophy and regeneration in the same animal would be highly desirable to reduce the number of animals in experimental studies on peripheral nerve regeneration and yet have an adequate statistical power.

Ultrasound has already been implemented at large as a clinical imaging tool for the musculoskeletal system. In experimental research it is also a widely used tool; an example is the assessment of muscle after denervation in a rabbit model. [10] Additionally, clinical studies have been published describing the ultrasonographic measurement of atrophy and muscle cross sectional area with a high reliability and with a strong correlation to MRI findings (i.e., ICC = 0.74–0.94) [11], [12].

Hence, the purpose of this study was to examine the reliability and validity of ultrasonographic measurement of gastrocnemius muscle size after denervation as an alternative to the GMI index. The following questions were investigated: (1) Is ultrasonography a valid measure of muscle atrophy when compared to muscle mass and (2) what is the interrater and intrarater reliability of ultrasonographic measurement of muscle atrophy. Our hypothesis is that non-invasive ultrasound assessment of the gastrocnemius muscle over time could be a reliable and valid alternative to the GMI that will allow repeated measurements in the same animal at different points in time without having to sacrifice the animal.

## Methods

### Animals and Anesthesia

Animals were cared for under the guidelines of our center and the experiment was approved by the Animal Experiments Committee of the Erasmus MC, University Medical Center (Permit number: DEC#133-11-03) according to the National Experiments on Animals Act and conducted following this law that serves the implementation of Directive 86/609/EC of the Council of Europe.

Sixteen male Wistar rats, weighing 250–300 gr, were studied. All animals were operated under general anesthesia (Isoflurane administered continuously via nose cone, 1–2% in O_2_). During the procedures, the animals were monitored by visual inspection (i.e., breathing) and the temperature was maintained by means of a computer-regulated heating pad (37°Celcius).

### Surgical Technique

The surgical procedure was performed by a single surgeon using standard aseptic techniques and an operating microscope (Zeiss OP-MI 6-SD; Carl Zeiss, Goettingen, Germany) on the sciatic nerve of the left hind limb. The non-operated right limb sciatic nerve served as pairwise control. The sciatic nerve was exposed through an oblique skin incision over the gluteal region. A muscle splitting approach was utilized to reach the nerve. An 8 mm segment of the nerve was isolated, placing sutures on both the proximal and distal nerve stumps before excising. After removing the nerve segment, the muscle was closed using 1 6/0 Vicryl Rapide suture, followed by closing the skin using 6/0 Vicryl Rapide Sutures (Ethicon, Johnson & Johnson, Amersfoort, the Netherlands).

### Experimental Set-up

**Figure 1:**
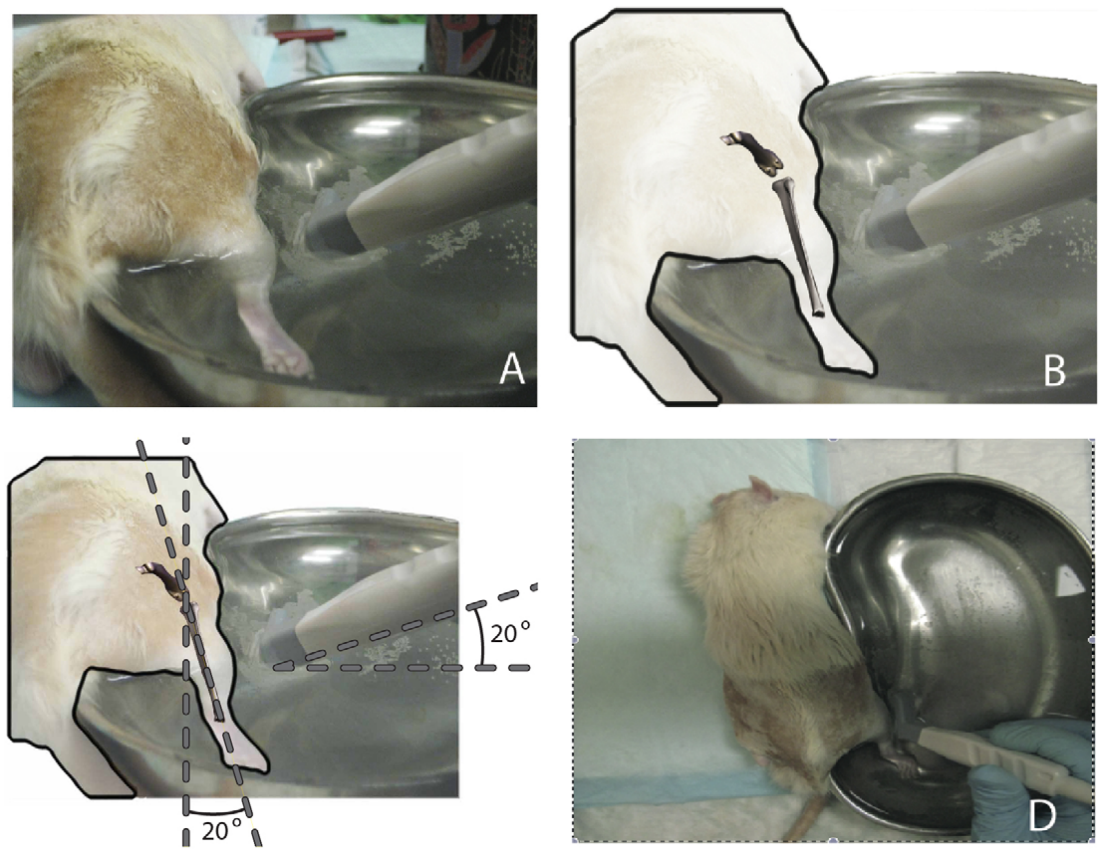
Experimental set-up. The experimental set-up of the ultrasound recording using the Philips L15-7io probe. (A) Position of the ultrasound probe in relation to the rodent, (B) illustration of the position of the tibial bone relative to the probe, (C) angulation of the probe (20 degrees in both the Z-Y axis) and (D) the position of the probe from above.

The rodents were examined at regular intervals for 2 months after the index procedure of creating the nerve defect in all sixteen rodents. During a 2 month period after creating the nerve defect in all sixteen rodents, each week, the gastrocnemius muscle in two animals was assessed by ultrasound and consequently the gastrocnemius muscle index was determined. For the ultrasound evaluation, the leg of the animal was placed in a stainless steel emesis basin that was filled with water as a substitute for conductivity gel and to standardize the pressure on the gastrocnemius muscle during the procedure. The rodent was kept warm using the heating pad and placed at the same level as the top of the emesis basin. The leg of the rodent was positioned at an angle of 20° to the basin side, with the toes on the floor of the emesis basin. The leg was positioned in such a way that the iliac crest of the leg was located on top of the basin edge. A detailed illustration of our experimental set-up is depicted in Figure 1.

### Ultrasound Recordings

Two investigators blinded to each other’s results performed the ultrasonographic examinations in the same session, both using two different ultrasound systems. The ultrasound recordings were performed with the SonoSite Titan Ultrasound (SonoSite Inc., Bothell, USA) and the Philips iU22, NZE 737 ultrasound system (Philips Healthcare – Ultrasound, Eindhoven, the Netherlands). The Titan SonoSite had a L38 probe (5–10 MHz) and the Philips system a L15-7io probe (7–15 MHz). The gastrocnemius thickness and cross-sectional area were determined by scanning in the sagittal plane of the muscle. In each measurement, the ultrasound probe was placed transversely on the gastrocnemius muscle at an approximate 20° angle from the water surface.

**Figure 2:**
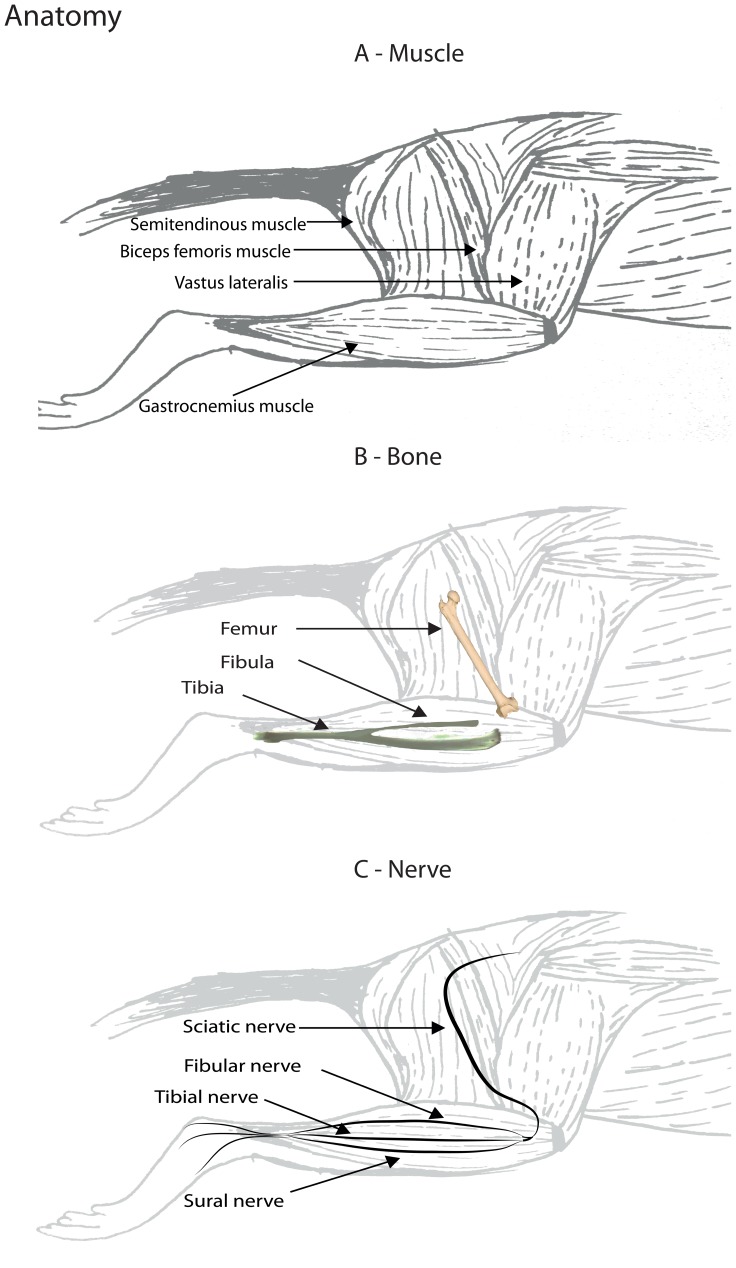
Anatomy. An overview of the anatomy of the rat hind limb. (A) Depicts the anatomy of the muscles, (B) shows the skeletal system and (C) illustrates the major nerves.

**Figure 3:**
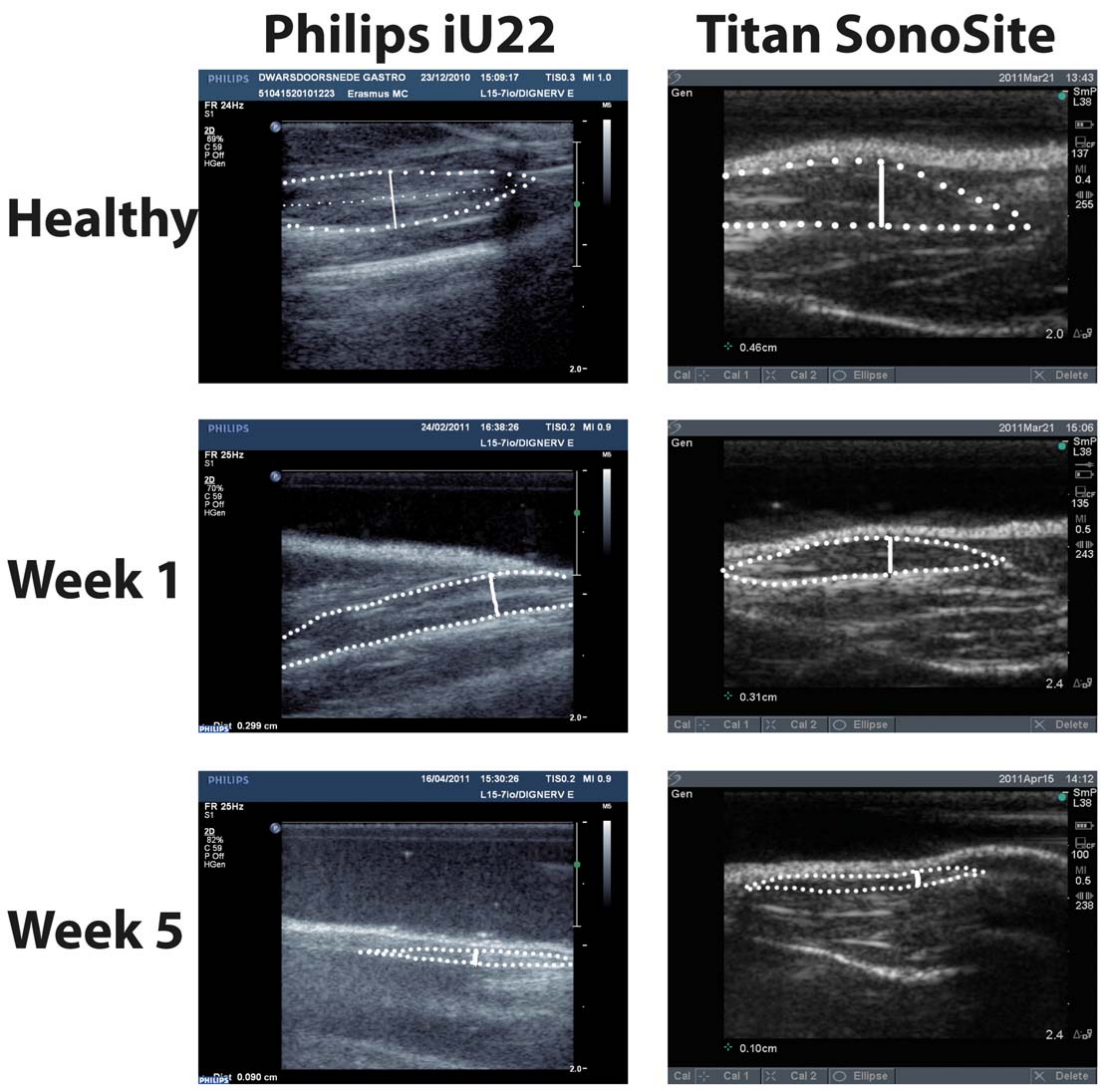
Ultrasound recordings. Serial ultrasound recordings with both the Philips iU22 and the Titan SonoSite of the gastrocnemius muscle after denervation. The thick interrupted line depicts the gastrocnemius muscle. The thin interrupted line depicts the peroneal nerve in the image of the healthy muscle recorded with the Philips iU22. The thick interrupted line illustrates the circumference of the gastrocnemius muscle. The non-interrupted line indicates the standardized muscle diameter measurement.

The location used to measure muscle thickness was standardized to find the most reproducible measure of the gastrocnemius muscle at, or close to, it’s thickest point (i.e., the maximum diameter). The standardized protocol consisted of identifying pre-determined landmarks: 1) the fibula, 2) the fibular nerve, and 3) the junction between the most distal point of the semitendinosus muscle and gastrocnemius muscle in the ultrasound image. An overview of the anatomy essential for identifying the landmarks is depicted in Figure 2. After identifying these three anatomical landmarks in the same ultrasound image, we measured the muscle thickness as the diameter of a line starting from the third anatomical point, perpendicular to the fibular nerve and the fibula bone. This procedure was repeated three times by each investigator and then averaged for further analysis. Figure 3 depicts typical examples of the gastrocnemius ultrasound recording and the anatomical points that were selected.

### Gastrocnemius Muscle Index (GMI)

Following the ultrasonography evaluations, the animal was euthanized by an overdosage of barbiturates administered intravenously. Consequently, the muscle was excised from the lower leg and wet muscle weight was measured immediately using a digital scale. The contralateral gastrocnemius was also harvested and the GMI was calculated by dividing the muscle weight from the operated side by that from the contralateral side.

### Data Analysis

A Pearson correlation coefficient was used to determine the relation between muscle weight and muscle thickness measured with ultrasonography. The inter-rater reliability of the ultrasound recordings was expressed for both ultrasound devices separately using an interclass correlation coefficient (ICC) and its 95% confidence interval (CI). To allow comparison of the ultrasonography data with the GMI, which is expressed in percentages as the ratio between the operated and the non-operated leg, we also expressed the muscle thickness as a ratio between both legs. To determine the difference between the GMI and the muscle thickness ratio between both legs, we used a Bland-Altman plot where the GMI was plotted on the x-axis and the difference between the GMI and the muscle thickness ratio was plotted on the y-axis. Significance was set at p<0.05.

## Results

The fibula, fibular nerve and the connecting point of the semitendinosus muscle fascia and gastrocnemius muscle could be unambiguously identified in all animals after a short learning curve. The average time for preparing the session was 5 minutes, identifying the three anatomical landmarks in a single image and measuring the diameter took about 10–15 min per leg.

**Figure 4:**
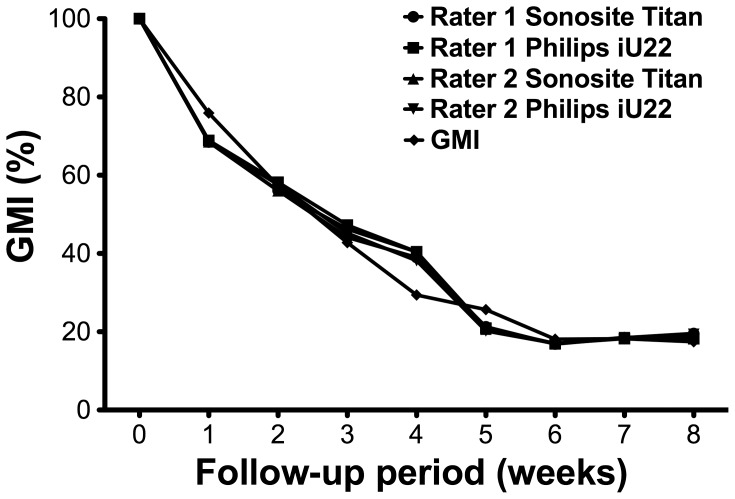
Decrease of muscle mass after denervation. The weight-based GMI and ultrasound-based muscle diameter in the operated leg expressed as a percentage of the non-operated side. To show the pattern without averaging, only the weekly data of the first animal are displayed.

Figure 4 illustrates the percentage of decrease of both muscle weight (GMI) and muscle thickness (i.e., diameter measured with ultrasound). A steep descent of muscle weight of 24% was observed as early as one week after the nerve defect was created. In the following weeks, the weight further decreased and then remained stable from 6 weeks onwards. A maximal decrease of the wet muscle weight of 82% was observed. Figure 4 illustrates the percentage of decrease of muscle weight (GMI) and of muscle thickness (i.e., diameter) measured with ultrasound. The ultrasound recordings had a highly similar curve of increasing muscle atrophy over time as the curve of the GMI decreased.

**Figure 5:**
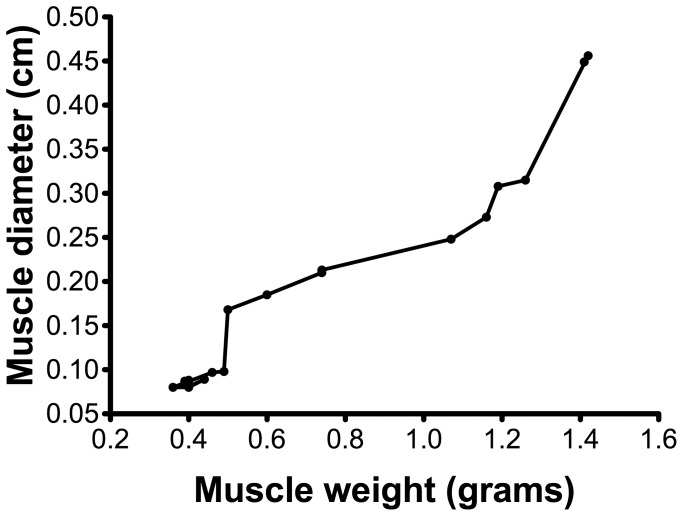
A scatter plot of the gastrocnemius muscle and the measured muscle diameter with ultrasound. Scatter plot of the gastrocnemius mass of the affected limb and the diameter of the muscle measured with ultrasound. Correlation coefficients ranged between 0,957 and 0.971 (p<0.001 for all) for both raters and both ultrasound machines.

Figure 5 illustrates the strong correlation between GMI and muscle thickness from a single rater using the SonoSite Titan for the operated leg, confirmed by a correlation coefficient of 0.97. The same was found for the Philips ultrasound measurements of the same rater, as well as for the measurements of the other investigator with both ultrasound devices (0.96 for all).

**Figure 6:**
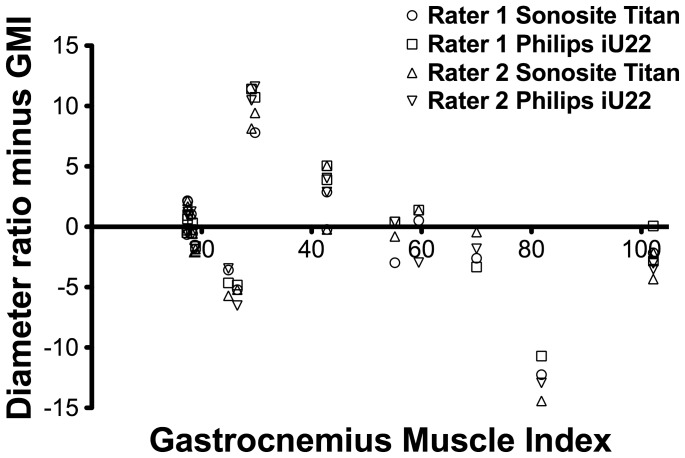
The difference between muscle diameter and the gastrocnemius muscle weight. A Bland Altman plot to visualize the difference between the muscle diameter measured and the gastrocnemius muscle weight (GMI) in percentage of atrophy. The GMI is plotted on the x-axis and the difference between the GMI and the muscle thickness ratio on the y-axis.

The interobserver reliability was excellent for both devices on the operated side (for both devices: ICC 0.99, 95% CI 0.98–.99) for both devices) and good for the non-operated site (ICC 0.84, 95% CI 0.62–0.94) for the Titan Sonosite and good (ICC 0.89, 95% CI 0.66–0.95) for the Philips iU22. The high similarity between GMI and ultrasound recordings was further confirmed by the Bland Altman plots (Figure 6), showing that both methods generally did not differ more than 5%, with two outliers of approximately 13%.

## Discussion

The need for serial evaluation of muscle re-innervation in a nerve injury model is imperative to determine short and long term outcomes after nerve reconstruction. This study revealed (1) a high correlation between muscle weight (GMI) and atrophy evaluated using ultrasound and (2) a high intra-rater and inter-rater reliability of the ultrasound measurements.

Previously, techniques available to evaluate serial nerve regeneration over time were limited to the sciatic foot index and the pinprick evaluation. [13] Other important measurements, such as the evaluation of muscle atrophy and electrophysiology, require invasive procedures that often necessitate sacrificing the animal [14], [15].

The rationale to use ultrasound in the examination of muscle atrophy has already been discussed in several studies, all revealing a successful visualization in both a non-pathological situation as well as after a nerve injury. [16], [17], [18], [19], [20], [21] In addition, Severinsen et al. demonstrated the usefulness of ultrasound for evaluating foot muscle atrophy in diabetic patients, suggesting that ultrasound is a reliable method to assess muscle atrophy caused by nerve injury. [12], [22] The high reliability of the ultrasound measurements reported in this study may largely be explained by the standardized protocol. This protocol requires a predetermined set of anatomical landmarks to enable the observers to repeatedly measure the same section of the muscle. Another factor contributing to the reliability of the method was measuring the gastrocnemius diameter under water. The application of a water bath surrounding the muscle creates an equal-pressure environment and allows an accurate assessment of the muscle, preventing the muscle from compressing during different measurements.

We recommend practicing this method before implementation in a study because of the learning curve of examiners. A number of practice sessions was necessary to establish a reproducible measurement protocol and also several sessions to teach the second investigator to identify the anatomical landmarks and to reproduce the measurements accurately. Our experience showed an initial assessment of approximately 30 minutes per animal while our final test recordings were completed within 15 minutes. We therefore advise to have at least 5 extensive training sessions.

A limitation of the study is the discrepancy between the muscle diameter measured using ultrasound and the GMI at weeks 4 and 5. While the ultrasound measurements did show a high correlation between the two investigators and ultrasound systems, a difference was found when compared to the GMI. A possible explanation could be that our ultrasound recordings are 2D, while the muscle weight more strongly correlates with the 3D shape of the muscle. It could be that the muscle atrophy leads to a situation where the 2D simplification is not as accurate as when there is no atrophy. Another possible explanation could be the muscle density, since the muscle diameter only gives an indication for muscle volume and not for density. The density is defined as the ratio between the cell volume of the muscle cells and the amount of collagen. As atrophy develops, the muscle cell volume decreases and the collagen deposit increases. In contrast, the GMI is calculated based on wet muscle weight and therefore includes both density and volume. Another limitation is that muscle volume is an indirect measure of re-innervation. Naturally, muscle force is a more direct measure of motor re-innervation. This limitation, however, applies to the commonly used GMI method as well. Only studying the muscle diameter and weight during the atrophic phase of the muscle-weight curve could cause the third limitation. While similar results may be anticipated, future studies are needed to establish if the same method can measure changes in muscle diameter during re-innervation.

These limitations notwithstanding, our data indicate the development of a reliable and sensitive technique to evaluate muscle diameter and thus to evaluate the amount of muscle atrophy. Although we only tested the gastrocnemius muscle, we are convinced that this method can be applied successfully for assessing other muscle groups as well, including smaller muscles. However, for each muscle, a well-defined protocol needs to be developed to assure that the muscle is measured at a repeatable location. This non-invasive technique is especially interesting since it can add to other minimally invasively techniques. We have recently demonstrated the possibility to record compound muscle activation potentials (CMAPs) without sacrificing the animal, allowing another measure of nerve function during regeneration in the same animal over time. [23] Taken together, these techniques allow reducing the number of sacrificed animals in future experiments and allow recording repeated data over time in the same animal when evaluating nerve regeneration.
