# Correction: A New Approach to Assess the Gastrocnemius Muscle Volume in Rodents Using Ultrasound; Comparison with the Gastrocnemius Muscle Index

**DOI:** 10.1371/journal.pone.0133944

**Published:** 2015-07-20

**Authors:** Tim H. J. Nijhuis, A. Siebe de Boer, Abhijeet L. Wahegaonkar, Allen T. Bishop, Alexander Y. Shin, Steven E. R. Hovius, Ruud W. Selles

The second author’s name is spelled incorrectly. The correct name is: A. Siebe de Boer. The correct citation is: Nijhuis THJ, de Boer AS, Wahegaonkar AL, Bishop AT, Shin AY, Hovius SER, et al. (2013) A New Approach to Assess the Gastrocnemius Muscle Volume in Rodents Using Ultrasound; Comparison with the Gastrocnemius Muscle Index. PLoS ONE 8(1): e54041. doi:10.1371/journal.pone.0054041
